# E-LEARNING FOR IMPROVING PERSON-CENTERED ATTITUDE IN PROFESSIONALS WORKING WITH PEOPLE WITH DEMENTIA: PILOT STUDY

**DOI:** 10.1093/geroni/igz038.3260

**Published:** 2019-11-08

**Authors:** Claudia van der Velden

**Affiliations:** Netherlands Institute of Mental Health and Addiction (Trimbos-institute), Department on Aging, Utrecht, Netherlands

## Abstract

Healthcare professionals working with people with dementia (PwD) have increasingly been moving towards a person-centered care (PCC) attitude. Several studies have showed positive results of PCC on quality of life of PwD. Also, it shows positive effects on self-esteem and work-satisfaction of professionals. An effective way to educate professionals in PCC is by using online-learning-tools. We developed an e-learning - in co-creation with end-users - focusing on well-being and challenging behavior of PwD. The interactive e-learning supports healthcare professionals in developing a PCC-attitude, using practical videos and exercises. In the current pilot-study, the e-learning is evaluated. To date, 32 professionals working in Dutch care homes from different care organizations participated in the study and completed the e-learning. In addition, they filled in online questionnaires before and after completing the e-learning. The questionnaires include the Approach to Dementia Questionnaire, Dementia Knowledge, Person Centered Care, Sense of Competence in Dementia Care Questionnaire. Post-measurement also included questions about satisfaction with the e-learning and user-friendliness. The final participants are currently completing their post-measurement questionnaires and final results are expected in October 2019. Preliminary data-analysis shows promising results. Although no significant effects were found, two-thirds of the participants indicated they had more knowledge about dementia, could better deal with challenging behavior and understood better how their own behavior and actions influence the behavior of PwD. The user-friendliness was also positively evaluated. Preliminary results suggest that the e-learning helps professionals in their approach to PwD and indicate that participants have positive experiences with the e-learning.

